# Discharge health education needs and experiences of patients with skeletal-related events from bone metastases: A qualitative study

**DOI:** 10.1371/journal.pone.0341790

**Published:** 2026-06-12

**Authors:** Jing Shan, Shuangyan Wang, Fan Ding, Junli Liang, Jianxin Wang, Donglai Wang

**Affiliations:** 1 Department of Orthopedics, The Fourth Hospital of Hebei Medical University, Shijiazhuang, Hebei, China; 2 Department of Nursing, The Fourth Hospital of Hebei Medical University, Shijiazhuang, Hebei, China; 3 Department of Breast Surgery, The Fourth Hospital of Hebei Medical University, Shijiazhuang, Hebei, China‌‌; University of Life Sciences in Lublin, POLAND

## Abstract

**Objective:**

To describe the health education needs and experiences of patients with skeletal-related events due to bone metastasis from solid tumours upon discharge and to provide a reference for the formulation of discharge health education plans for this population.

**Methods:**

A qualitative descriptive design and purposive sampling method were used to select patients with skeletal-related events due to bone metastasis from solid tumours who were hospitalized in the orthopaedic department of a Class III Grade A cancer hospital in Hebei Province from June to July 2024 for in-depth semistructured interviews. A Colaizzi 7-step analysis method was used to refine the themes of the patients’ needs.

**Results:**

The needs of solid tumour bone metastasis patients with skeletal-related events for discharge health education provided by medical staff were refined into five themes: 1) bone health knowledge needs: patients were eager to obtain in-depth knowledge beyond the definition of the disease, such as the specific warning of pathological fracture and the early identification of bone metastasis progression; 2) bone health self-management needs: the focus was on specific operation guidance, including the home application of analgesic stepwise therapy and the protection skills for bone destruction sites; 3) coping with daily life needs: patients were concerned about how to safely go to the toilet, take a bath and other daily activities under the risk of disability; 4) coping with psychological pressure needs: manifested as the counselling needs for fear of disability and psychological support to combat the “burden feeling” during long-term pain; and 5) family and social support needs: patients need a collaborative nursing model that includes family participation, as well as a remote consultation channel for continuous access to professional medical care after discharge. Three themes were extracted from the information experience: 1) barriers to understanding medical terms: the professional words used by medical staff (such as “skeletal-related events” and “bone scan”) confused patients, leading to incorrect interpretation or neglect of health education; 2) passive acceptance: patients were unable to obtain targeted information, and the existing education is mostly one-way indoctrination, which fails to consider the differences in personalized care after bone metastasis of different primary cancers (such as lung cancer, breast cancer and prostate cancer); and 3) the large amount of information leads to poor satisfaction: the large amount of fragmented data provided before discharge leads to information overload and cognitive fatigue among patients, and the actual mastery rate and application satisfaction after discharge are low.

**Conclusions:**

Patients with skeletal-related events due to bone metastasis from solid tumours face challenges such as a lack of bone health knowledge, psychological distress and a lack of self-management ability when discharged from the hospital. Medical staff should formulate a personalized discharge health education plan according to the needs of patients.

## Introduction

Up to 89% of prostate cancer patients, 75% of breast cancer patients and 40% of lung cancer patients with advanced solid tumours will develop bone metastasis during the course of the disease [1], and bone metastasis is among the important causes of severe pain in patients with advanced cancer. It also increases the risk of skeletal-related events (SREs), which increase the risk of death and medical expenses and seriously affect the quality of life of patients [2,3]. Bone health is a prerequisite for patients to have the ability to live independently and return to normal work status and is closely related to quality of life. Patients with bone metastasis from solid tumours have multiple symptoms, complex disease treatment, and uncertain disease development. Under the Diagnosis-Intervention Packet (DIP), a medical insurance payment system based on disease score in China,) policy [4], the length of hospital stay of patients is shortened. As a result, most patients do not fully recover and need home rehabilitation and self-management. When discharged, patients face the following challenges: a lack of bone health knowledge, psychological distress, insufficient belief, and the inability to perform tasks such as exercise, daily life tasks, medication administration and regular screening after discharge [5]. Therefore, improving self-management ability, coping responses, and knowledge about bone health before discharge are urgent priorities.

Studies have shown that the incidence of SREs in patients with breast cancer, lung cancer and prostate cancer is as high as 63%, 59% and 52%, respectively [6]. Patients with untreated bone metastases experience SREs every 3–6 months on average [7]. Therefore, it is very important for patients with solid tumour bone metastasis and SREs to adhere to regular treatment with bone protective agents. Patients at home should continue to take calcium and vitamin D supplements according to the doctor’s prescription, change poor lifestyles, supplement nutrition, avoid falls, actively report treatment effects and adverse drug reactions, and regularly return to the hospital for monitoring and evaluation of bone health parameters [3] to delay the occurrence of SREs or reduce the frequency of SREs. Research on the information needs of patients with advanced cancer has shown [8–10] that patients need information about preventive and treatment measures, self-management skills and coping strategies. Patients hope to acquire relevant health knowledge and self-management skills and improve self-efficacy before discharge. The time prior to discharge is considered the critical period for health education for patients and caregivers [11]. Health education at discharge is particularly important. Bone health knowledge and self-care can be improved by providing discharge education [12]. Health education information is crucial for patients’ participation in medical care and self-management tasks and improving their medical experience [13]. Medical staff can improve the treatment compliance and self-management ability of patients after discharge, eliminate or reduce the risk factors affecting health, and reduce the rehospitalization rate through health education [14,15]. In addition, emotional support can be given to patients with SREs in advanced solid tumours to help them adopt positive coping styles after discharge, promote mental health, and establish beliefs to overcome the disease [16,17]. In summary, understanding the specific needs and experiences of patients is essential for health education after discharge.

Compared with quantitative research limited by closed questions, qualitative research has unique advantages in exploring the complex life experiences of patients with SREs among patients with solid tumour bone metastasis. Discharge education for patients with SREs involves not only knowledge transfer but also patients’ adaptation to role changes and their ability to cope with the fear of death in the real-world environment. These large information blind spots are difficult to capture by traditional questionnaires. In this study, a phenomenological interview method was used to explore the subtle and emotional experiences of patients with SREs during the transition period of discharge to reveal the needs and essential logic behind the complex difficulties after discharge. The core goal of this study is to compensate for the limitations of quantitative research in explaining “why” and “how” and to provide a solid empirical basis for developing evidence-based, patient-centred, and culturally sensitive personalized discharge health education plans.

## Objects and methods

With the use of the purposive sampling method, maximum variation sampling based on patient characteristics (sex, age, education level, disease type, and duration of bone metastasis) was used to select patients with solid tumour bone metastases and SREs who were hospitalized in the Department of Orthotopic Surgery of a Class III Grade A cancer hospital in Hebei Province from June to July 2024 as the research objects. The inclusion and exclusion criteria are shown in Table 1. The sample size in which no new coding or subthemes appeared in the data analysis was the sample size selected for the interview. Real-time transcription was performed during each interview, and three-level coding was performed after each interview was completed. After the 15th interview (P15) was completed and cross-analysed by two researchers independently, a team consensus meeting was conducted to confirm that no new primary coding or meaning construction had occurred. To further verify the completeness of the information, in accordance with the prudent principle of qualitative research, 3 additional patients with different backgrounds were interviewed (P16–P18). The results revealed that the additional 3 patients supplemented only details that fell into the existing themes, and no new third-level themes were generated. Therefore, the sampling was ultimately stopped, and a total of 18 patients were included. The data saturation analysis table is shown in Table 2. The general information of the respondents is shown in Table 3. The ages ranged from 40 to 71 years, with a mean age of 55.78 ± 11.46 years, and the duration of bone metastases ranged from 5 to 72 months, with a mean of 20.06 ± 15.45 months. This study strictly followed the Declaration of Helsinki and was approved by the Ethics Committee of a tertiary cancer hospital in Hebei Province (No. 2022KY090). In the informed consent process, the investigators first made initial contact with the potential subjects and their families and explained the purpose of the study, interview content, recording requirements, and privacy protection measures in plain language. To ensure the full consideration of the subjects, the researchers gave them at least 24 hours to contemplate their participation in the study after the initial contact, after which the subjects or their legal guardians signed the written informed consent form. No subjects refused to participate or withdrew from the study. For the vulnerable group of patients with SREs in advanced solid tumours in this study, the following safeguards were taken: 1) Protection of the right to know: Researchers verified the awareness of the subject's condition again before the interview to ensure that the interview process did not break through the scope of the patient's known condition. 2) Physical and mental health monitoring: During the interview, researchers paid close attention to the physical conditions (such as pain and fatigue) and emotional fluctuations of the subjects. If the subjects experienced obvious discomfort or psychological stress, the researcher immediately suspended or terminated the interview and provided necessary psychological counselling or medical assistance. 3) Right to voluntary withdrawal: Subjects were clearly informed that their participation was completely voluntary and that they had the right to withdraw their data at any stage of the study (including after the interview) without prejudice to the quality of their subsequent treatment and care.

**Table 1:** Inclusion and Exclusion Criteria.

Category	Detailed Criteria
Inclusion Criteria	1. Primary malignant tumour confirmed by pathology
	2. Bone metastases with skeletal-related events (SREs) confirmed by imaging
	3. Age ≥ 18 years old
	4. ≥ 2 hospitalizations
	5. Good language expression and communication skills
	6. Be aware of the condition
Exclusion Criteria	1. Bone metastases caused by haematological malignancies
	2. Severe mental disorder or cognitive impairment
	3. Be critically ill or have serious complications of other systems

**Table 2:** Data Saturation Analysis Results.

(Interview No.)	(Key Characteristics)	(Examples of New Codes Emerging)	(Saturation Status)
P1 - P5	Different types of cancer, age, and sex	Medical concealment (family protection), coping with psychological stress, medication confusion, passive acceptance of information, and obscure information terms	The period of rapid growth of coding
P6 - P10	Different courses of disease and educational backgrounds	The needs of bone health knowledge, the needs of daily life, the fear of activities, the desire for targeted answers, and the confusion of rushed discharge	Coding continued to increase
P11 - P15	Cover different health insurance types	The need for remote consultation and the desire for professional guidance	No new codes appeared after the 15th case
P16 - P18	Confirmatory sample	None (No new codes emerged)	Confirmed as theoretical saturation

**Table 3:** General Information Sheet of respondents.

Number	Sex	Age (years)	Education	Marital status	Occupations	Place of residence	Type of health insurance	Type of illness	Duration of bone metastases (months)
P1	Male	69	Junior college	Married	Retired	Towns	Town employees	Bone metastasis of oesophageal cancer	22
P2	Male	68	High school	Married	Retired	Towns and cities	Town employees	Bone metastases from colon cancer	26
P3	Female	65	Elementary school	Married	Worker	Rural	Urban and rural residents	Renal cell carcinoma with bone metastasis	6
P4	Male	66	Undergraduate	Married	Retired	Towns	Town employees	Bone metastasis of prostate cancer	19
P5	Female	40	Junior high	Married	Farmer	Rural	Urban and rural residents	Lung cancer bone metastases	11
P6	Female	60	Elementary school	Married	Farmer	Rural	Urban and rural residents	Bone metastases from breast cancer	29
P7	Male	55	Junior high school	Married	Farmer	Rural	Urban and rural residents	Lung cancer bone metastases	20
P8	Male	45	Undergraduate	Married	Individual	Town	Town employees	Bone metastases from lung cancer	7
P9	Male	43	Master's	Married	Staff member	Town	Town employees	Gastric cancer bone metastasis	8
P10	Male	67	High school	Married	Retired	Towns	Town employees	Bone metastases from lung cancer	16
P11	Male	42	Master's	Married	Clerk	Town	Urban workers	Bone metastases from lung cancer	23
P12	Male	52	Undergraduate	Married	Teacher	Towns	Town employees	Bone metastases of rectal cancer	28
P13	Female	59	Junior high	Married	Farmer	Rural	Urban and rural residents	Bone metastases from breast cancer	27
P14	Female	45	Undergraduate	Divorced	Clerk	Town	Town employees	Gastric cancer bone metastasis	6
P15	Female	48	Junior high	Married	Farmer	Rural	Urban and rural residents	Bone metastasis of thyroid cancer	72
P16	Male	40	High school	Unmarried	Clerk	Town	Town employees	Bone metastases from colon cancer	24
P17	Male	71	Primary school	Married	Farmer	Rural	Urban and rural residents	Bone metastasis of oesophageal cancer	12
P18	Male	69	Primary school	Married	Farmer	Rural areas	Urban and rural residents	Lung cancer bone metastases	5

### Research methods

#### Determine the interview outline.

In accordance with the purpose of the study, the first draft of the interview outline was developed through a literature review and research group discussion. Before the formal interview, the research team conducted a preinterview with 3 patients who met the inclusion criteria to evaluate the applicability of the outline. On the basis of the results of the preinterview and expert consultation, the research team made the following key revisions to the first draft: 1) Language popularization: the medical terms in the original outline (such as “prevention of pathological fractures” and “self-monitoring of SREs”) were too abstract for some subjects. After revision, it was changed to a more concrete description, such as “Do you know what conditions may indicate that a bone is at risk of fracture?” “And” How do you observe the condition of your own bones? “To ensure that different subjects understood exactly what it meant. 2) Adjusted the order of the questions: The preinterview revealed that asking directly about “needs” may be difficult for the subjects. After the revision, open topics such as “feelings” and “life impact” were introduced to help the subjects enter the situation, and then “urgent needs” and “confusion” were further discussed. 3) Addition of introductory words: In view of the phenomenon that the subjects’ answers were brief in the preinterview, auxiliary hints were added to the outline. For example, when asking about “life impact”, prompts about specific life scenes such as eating, toileting, and turning over were added to induce deeper information output. See Table 4 for the final interview outline.

**Table 4:** Interview Outline.

Domain	Interview Questions
Current Experience (Current Experience)	1. Could you talk about your overall experience of the discharge health education you received before discharge?
Preferences	2. When and in what way do you think discharge education will be most helpful to you?
Core Needs	3. What guidance would you like to receive before discharge to manage your bone health? What do you feel is the most urgent need?
Impact on Life	4. What specific impact do you feel your current physical/medical status has had on your daily life after discharge?
Support Channels	5. What channels or platforms would you like to use for ongoing professional help and guidance after discharge?
Unresolved Concerns	6. What confusion or concerns do you have about managing your bone health after discharge?

#### Data collection methods and quality control.

Semistructured interviews were used to collect the data. The interviews for this study were conducted by the first author. The investigator is a female with a master's degree in nursing who is engaged in oncology nursing and has more than 20 years of clinical experience in nursing bone cancer patients. Face-to-face interviews were conducted in a classroom in an orthopaedic ward, with only researchers and patients present. The trusting relationship established between the researcher and some of the respondents during their hospital stay helped to establish the interview atmosphere, but it may also introduce potential personal bias. To manage this bias, researchers took the following measures: 1) the suspension method: Researchers implemented “suspension” throughout the entire process of the study. By writing reflective diaries, researchers identified and temporarily set aside their own preset tendencies as senior clinical nurses for discharge education and ensured that they listened to patients as external observers rather than guiding them as managers. 2) Neutral guidance: During the interviews, the researchers wore casual clothes instead of work clothes to reduce the sense of doctor‒patient hierarchy and reduce the respondents’ social expectation bias (providing positive feedback to please nurses). All the questions strictly followed the open-ended outline, avoiding the use of guiding words with clinical hypotheses. 3) Research team consensus: Data analysis was performed independently by two investigators, and the initial coding was reviewed by other members who were not involved in clinical care through team discussion during the topic refinement phase to correct possible empirical interpretations by the first author and ensure that the findings were always rooted in the original data. Each interview lasted 30–60 minutes. After the interview, the results were verified and revised to the respondents.

#### Data analysis methods.

The recording was transcribed into text within 24 hours after the interview and supplemented with onsite notes such as expressions, body movements and other nonverbal information. To ensure the reliability of the analysis, investigator cross-checking was used in this study; two researchers (the first author and another qualitative research member) independently coded the 18 transcripts at three levels. After coding, more than 140 initial codes generated were compared one by one by both parties. Fifteen percent of the conflicting codes, which concerned mainly the boundary between information experience and health needs, were discussed in weekly research team meetings, with an author with extensive experience in qualitative research as an arbiter, and 100% consensus was reached on all topic attribution.

Colaizzi's 7-step analysis method [18] was used to refine the themes. The steps were ① Read the interview records carefully and repeatedly; ② Extract important statements; ③ Encode recurrent and meaningful ideas; ④ Pool the coded viewpoints to form thematic groups; ⑤ Define the theme separately; ⑥ Comprehensive description of the phenomenon; and ⑦ Return the results to the respondents for verification and modify and supplement the results according to the feedback. In step ⑦, five representative respondents were selected (P1 male, 69 years old, 22 months old, oesophageal cancer with bone metastases; P6 female, 60 years old, breast cancer with bone metastases; P7 male, 55 years old, lung cancer with bone metastases; P12 male, 52 years old, rectal cancer with bone metastases; P15 female, 48 years old, thyroid cancer, bone metastasis) (covering different sexes and primary cancer types) The eight themes and their descriptions were presented to the participants by telephone. The feedback results revealed that all five respondents thought that the themes accurately expressed their true mood at discharge. Among them, P7 proposed that the description of “toilet safety” in “coping with the needs of daily life” should be more prominent, and the research team supplemented the detailed content of “guidance on fall prevention facilities”. This study focused on rigorous deduction from raw data to abstract topics. For example, for the theme of “self-management needs”, the researchers first extracted significant statements such as “I do not know how to take painkiller when I go home” and “I'm worried about breaking my bone when I move” from the original quotes and then coded them into “medication confusion” and “activity fear”. Finally, the topic group was grouped into “bone health self-management needs”.

## Results

### The needs of health education at discharge

#### Need for bone health knowledge.

Many respondents expressed that they wanted to obtain relevant knowledge to protect bone health after discharge and were not sure what factors harm bone health and what factors can promote bone health. “I have had this breast cancer for so many years, so I have a good understanding of it, but I do not understand the knowledge of bone metastasis, and I do not know how to make bones healthier!” (P6, female, 60 years old, breast cancer bone metastasis, 29 months). “I will be discharged from the hospital tomorrow. I have broken a bone once. I hope the professional medical staff will tell me some little knowledge about how to keep my bones healthy, or I have no knowledge at all.” (P4, male, 66 years old, prostate cancer bone metastasis, 19 months).

#### Bone health self-management needs.

Before discharge, some respondents hoped to engage in self-management behaviours to maintain bone health, such as sports and exercise at home, strategies to prevent falls, when to return to the hospital for bone health examination, and precautions for medication. “I still have some simple work to do after I return home, and I do not know if I can do it. I used to have the habit of walking and practising Tai chi, but this time I had a bone problem in the hospital. If there were professional people to give me more guidance in this aspect!” (P12, male, 52 years old, bone metastasis of rectal cancer, 28 months). “I know I need to maintain proper activities after discharge, but I am worried about falling and what to pay attention to when exercising, and I want to get guidance on this.” (P9, male, 43 years old, gastric cancer with bone metastasis, 8 months). “How often I should be reexamined after discharge from the hospital, and I do not know what to check, what to pay attention to the calcium I use and the drug to protect my bones. I hope that someone will pay attention to and remind me and answer my confusion.” (P4, male, 66 years old, prostate cancer bone metastasis, 19 months).

#### Coping with the demands of daily life.

After they returned home, the respondents wanted to receive guidance on diet and daily habits. “I have the habit of smoking and drinking. For so many years, I think smoking is definitely not good for me to be hospitalized for bone metastasis. After discharge, I will not smoke. (P1, male, 69 years old, oesophageal cancer with bone metastasis, 22 months). “This time I have to take a good supplement and add more supplements or nutrients, which will definitely be good for my bones!” (P11, male, 42 years old, lung cancer bone metastasis, 23 months). “What I am most worried about is now that the spine will press against the nerve. I am afraid that if I am not careful, I will not be able to stand up and move. The most important thing I care about every day is how to sit on the toilet, how to turn over when sleeping, and how to move safely at ordinary times. I am afraid that the action will not hurt the bone or aggravate the condition. I do not care about anything else. I just want to know how to do the most basic and practical daily movements so that I can take care of myself safely and not add big trouble to my family.” (P7, male, 55 years old, lung cancer bone metastasis, 20 months).

#### The need to cope with psychological stress.

Most of the respondents expressed that they were worried about the progression of the primary tumour and the recurrence of skeletal-related events. In addition, the fear of pain and surgery caused a great psychological burden for the respondents, and they urgently needed effective coping methods. “I have no strength after this leg surgery, I cannot walk, it hurts when I move, I take painkillers, it does not work, how to take this medicine, I will be discharged tomorrow, I can do this ah, go back to rely on the children to take care of, my mind pressure can be big, just want to sleep, simply fall asleep and never wake up!” (P3, female, 65 years old, renal cancer with bone metastasis, 6 months). “This is the third time I have broken a bone because of my colon cancer, which is really painful. It is worse than once. Now I have to need someone else to turn over, how to go to the toilet, and trouble others for everything.” (P2, male, 68 years old, bone metastasis of colon cancer, 26 months).

#### Need for family and social support.

Some of the respondents were willing to participate in community activities after discharge, but they were worried and retreated. Some of the respondents were willing to share their feelings with family or friends for support and understanding, but ultimately, most of them chose to endure or be silent. “I'm running out of time, day by day. Although I do want to go to parties and such, I think to myself, what's the point? I have cancer, and people are even worried that I might infect them!” (P6, female, 60 years old, breast cancer bone metastasis, 29 months). “I have not had a video call with my son for a long time, and he does not understand it. Maybe just enlighten and enlighten, so that I can feel good for a few days!” (P15, female, 48 years old, thyroid cancer bone metastasis, 72 months). “I do don't want to say it to my children. I'm afraid I'm dragging my children down. They all have their own families and jobs, and they are already under great pressure. If I tell them too much about the disease, it will only make them worry, spend money and work hard. Therefore, I try to hide a lot of discomfort, say less, do not want them to think that I am a burden, in fact, I want to keep a little decent, do not need to be special treatment, be pitiful, can someone respect me, understand my age as a father, as a family, so that I can like a normal person, have their own dignity.” (P1, male, 71 years old, oesophageal cancer with bone metastasis, 12 months).

### Experience of health education at discharge

#### Barriers to understanding medical terms.

Many respondents said that the medical terms or jargon mentioned in the discharge education provided by medical staff made them feel confused and lost. They complained that the explanations given by medical staff were too professional and difficult to understand and hoped that plain and easy-to-understand language could be used instead of obscure medical terms. “When I was discharged from the hospital, I was told that I was going to take a drug at home, which seemed to be called some targeted drug. I was told that the bone might be necrotic after the drug was used, and I could not remember it!” (P2, male, 68 years old, bone metastasis from colon cancer, 26 months). “The doctors are very professional, speaking medical terms and informative; I actually didn't understand a lot of things. But I always feel that the doctor is the authority, we as patients have to listen, dare not ask questions, do not dare to express their confusion, for fear of being said not sensible, afraid of being troubled. Even if there are doubts and concerns, most of them are suppressed in the heart and do not dare to say more, and can only bear it silently “(P5, female, 40 years old, bone metastasis of lung cancer, 11 months).

#### Passive acceptance, unable to obtain targeted information.

Some respondents said that the medical staff taught them more precautions before discharge, but some of the respondents did not find this information useful. Some respondents said that they received a lot of information before discharge. However, not all the information could meet their actual needs. After discharge, knowledge and self-management behaviours related to bone health maintenance, such as the use of bone protective agents, daily lifestyles, exercise, and regular and timely follow-up, remain unclear. “The day before and on the day of discharge, the doctors and nurses gave me a lot of verbal information. I didn't really want to hear it. At first, I paid attention, but then I felt useless and embarrassed to interrupt!” (P2, male, 68 years old, bone metastasis from colon cancer, 26 months). “I especially want to know when I can go to the ground and do tai chi, but I'm not sure if my bone will be OK after the nail surgery?” (P8, male, 45 years old, bone metastasis from lung cancer, 7 months). “What should be paid attention to when using denosumab as a protective agent? I heard that some people have necrosis of the chin. I am quite worried, and I do not know how to prevent it or how to check it! I am old, the original memory is not good, plus the sick brain reaction is slow, before discharge from the hospital doctors and nurses all the things to pay attention to all finished, said fast and much, I was confused, a lot of did not understand. It felt like they were in a hurry and wanted to speed up turnover, regardless of whether I could accept it or not. I was discharged in such a hurry, and I had no idea how to take care of myself and how to deal with problems. My experience was really bad.” (P13, male, 69 years old, lung cancer bone metastasis, 5 months).

#### Large amount of information and poor satisfaction.

At the end of the interview, some respondents reported that they had received a lot of discharge health education information, but they still had insufficient confidence in maintaining or managing their own bone health after discharge, and their satisfaction with discharge health education was poor. “The nurse told me a lot of knowledge and methods about how to make bone health on the day of discharge, but I can't remember it. I'm confused and tired, and I'm sorry to interrupt her!” (P2, male, 68 years old, colon cancer bone metastasis, 26 months). “I don't want to hear a lot of talk to Rory before discharge. I want to meet problems after discharge and communicate with my doctor in charge and solve them in time. It's best to have wechat or phone. (P14, female, 45 years old, gastric cancer with bone metastasis, 6 months). “I have just been in hospital for a few days, and my body has not recovered much, when the doctor said that I can arrange to leave the hospital. I feel very rushed. On the day of discharge, the nurse told me a lot of precautions, how to take medicine, when to review, and what happened to come back as soon as possible. The information was so much that my mind was blank and I couldn't remember it at all. Obviously I was already very uncomfortable, the spirit is not good, but also in such a short time to listen to so much content, feeling that the hospital is in a hurry to catch up with the schedule, the heart is not quite the taste, also feel very uneasy, if can have a wechat group contact it would be good.” (P10, male, 40 years old, bone metastasis of colon cancer, 16 months).

## Discussion

### Correlation analysis between health education needs and information experience at discharge

This study revealed that the relationships between health education needs and the actual information experience of patients with solid tumour bone metastasis and SREs are reflected mainly in the following three dimensions:

#### Structural contradiction between high expectations of information content and low levels of acquired experience.

According to the results of this study, was great, and multidimensional information requirements, covering not only bone health knowledge but also more complex self-management and social psychological adaptation were lacking. However, patient experiences were characterized by “passive acceptance” and “insufficient targeting” (theme Passive acceptance, unable to obtain targeted information), and the reasons (e.g., P2, P8, P13) were related to the medical staff’s tendency to provide standardized and task-oriented discharge health education. Blanch-Hartigan et al. [19] reported that when health education fails to address the most urgent needs of patients, patients experience decision-making dilemmas, leading to low self-management efficacy after discharge.

#### Cognitive overload caused by high density and high barriers to information transmission.

Patients who were in need of hospital information reported that their experience was “informative”, however they also reported “poor satisfaction” (topic Large amount of information and poor satisfaction) and that the medical terminology was difficult to understand (Barriers to understanding medical terms), which embodies the cognitive load theory as it has a negative effect on the spread of health knowledge [20]. Owing to long-term cancer pain and surgical trauma, the physical and psychological resilience of patients with SREs is close to the limit (such as P2, P3, P10, and P14). At this critical turning point before discharge, medical staff relay a large amount of information through verbal means, which lowers the patients’ ability to engage in cognitive processing. Although medical terms further exacerbated information loss, patients obtained information from the desire to retreat, eventually leading to a dramatic decrease in the degree of patient satisfaction.

#### Conflict between the concealment of psychological support needs and the unitary health education model.

Patients had deep psychosocial support needs (theme The need to cope with psychological stress and Need for family and social support) when facing the fear of fracture and “burden”, but the existing information experience was mostly a one-way infusion of passive acceptance (theme Passive acceptance, unable to obtain targeted information). Owing to the lack of a two-way interactive feedback mechanism, patients’ psychological pain and support needs are overshadowed by complicated medical instructions. Some studies have shown [8] that health education lacking cultural sensitivity and emotional resonance aggravates the “information island” effect of patients, making them often choose to tolerate or be silent when facing the physical and mental damage caused by bone metastasis, which weakens the utilization of their social support system.

### Hierarchical analysis of the needs and experiences of groups with different characteristics

In this study, through analysis and comparison, the requirements for discharge education information and experience of the respondents were determined on the basis of sex, education level and the different characteristics of primary lung cancer.

First, in terms of sex, there are differences in the expression of psychosocial support needs between male and female patients. In this study, female patients (e.g., P6) were more inclined to express concern about social isolation and infection misunderstandings and were keen to join social groups such as patient self-help groups to experience emotional resonance. In contrast, male patients (e.g., P1) showed a stronger trait of forbearance and were more concerned about the loss of family responsibilities, and their need for psychosocial support was often reflected in the maintenance of self-esteem in the sick role. This study is consistent with that of Becque et al. [21], namely, that cancer supportive care should account for the sex-specific differences in coping mechanisms.

Second, there are differences in information understanding and acquisition efficiency among patients with different education levels. The patients with higher education levels understood the medical terminology better than patients with lower education levels (Barriers to understanding medical terms); the information was scientific and logical, however, it also had more stringent requirements, indicating a tendency towards active access to information. The respondents with lower education levels experienced severe cognitive overload (theme Large amount of information and poor satisfaction) and were more likely to distrust medical staff because of crude medical terminology (e.g., P13). Health literacy is a core variable that determines the equity of health education [22], and the differences observed in this study further confirm the urgency of developing visualization tools such as graphics, texts, and videos for low-literacy groups.

Finally, the difference between primary cancer and bone metastasis directly determines the priority of health education needs at discharge. Patients with a longer course of disease, such as breast cancer or prostate cancer (such as P6 and P4), have a more persistent need for long-term bone health management. In patients at risk of spinal metastasis accompanied by nerve compression (e.g., P7), the core focus is was toilet safety and the performance of specific daily life tasks. Such needs stratified on the basis of physiological characteristics suggest that standardized discharge health education must be transformed into a personalized path on the basis of clinical subtypes, which is consistent with the findings of Zhang et al. [23].

### Influence of cultural background and medical policy on discharge health education

First, in traditional doctor–patient interactions in China, doctors are often regarded as absolute authorities. This power imbalance leads interviewees to tend to remain silent when facing obstacles in understanding medical terms or a large amount of information (e.g., P2, P5) rather than actively interrupting or questioning medical staff. This obedient cultural background explains why patients still show passive acceptance despite poor experience, which also suggests that medical staff need to shift from simple knowledge indoctrination to a more interactive and empowering education model [24].

Second, China's core familism profoundly affects patients’ psychological needs and access to support. The interviews revealed that some patients (such as P1) chose to hide the details of their illness for fear of increasing the burden on their children or because of the “burdensome feeling”. In traditional Chinese culture, illness is often seen as an event for the whole family, and family members (especially children) play a dominant role in decision-making after discharge [25]. Therefore, health education at discharge should not only target patients themselves but also integrate the family support system and take the synchronous education of family members as the key to relieving psychological pressure and improving patient compliance.

Finally, the current healthcare policy environment, which includes the DIP policy, may indirectly exacerbate information overload. Under the DIP policy, the shortening of hospital days and the acceleration of bed turnover (e.g., P10, P13), as well as the abovementioned rush to discharge, force medical staff to focus a great deal of information and education within a very short discharge window. This time pressure conflicts with the cognitive overload of patients in the advanced stage of the disease, leading to poor patient satisfaction, which is consistent with the findings of the research of the Chinese scholar Zhu et al. [26].

### Construction of a “body-mind-belief” personalized discharge health education plan for patients with bone metastasis of solid tumours

In view of the complex health needs and information experience of patients with solid tumour bone metastasis and SREs, medical staff should formulate patient-centred and multidisciplinary personalized discharge health education, covering the following core dimensions:

Integrated psychosomatic medicine: implementing multidimensional pain management and referral mechanisms. Studies have shown that patients with SREs commonly suffer from severe psychological pain and suicidal thoughts caused by impaired function [27]. Therefore, discharge planning should break out of single health guidance and incorporate routine psychological distress screening (e.g., using the NCCN distress thermometer). For those with moderate to severe psychological burden, a clear referral pathway should be established for the psychological department, and psychologists should be invited to participate in planning [28]. Through the integration of family support, continuous care and peer education, we can alleviate the “burden” caused by the decline in the self-care ability of patients and improve their psychological resilience.

Optimization of communication efficiency: application of auxiliary tools to bridge the medical terminology gap. In view of patients’ barriers to understanding medical terms (such as “bone-protective agents” and “targeted therapy”), medical staff should abandon “cramming” propaganda and education. Communication-assisted tools can be introduced in clinical practice, such as problem prompt lists, visual picture manuals or metaphorical oral summaries, can be introduced to achieve the popularization of terms [29], such as changing “bone protective agent treatment” to “protective drugs for bone reinforcement”; at the same time, it is recommended that the “team-back method” be used to verify the patient’s understanding in real time. At the end of the education, patients are asked to repeat in their own language: “If you come home and find that your legs suddenly have no strength, what should you do in the first place?” Information forgetting and anxiety should be reduced to improve treatment compliance and reduce the risk of refracturing.

Relying on information platforms: To address patients’ desires for bone health support information after discharge, a “hospital–community–family” continuous support system should be built with the help of mobile applications (apps) or electronic information platforms [30] to ensure that patients can obtain immediate professional guidance and psychological support for bone health during the critical transition period and ultimately improve their quality of life and prolong their survival time.

### Implementing a personalized discharge education checklist on the basis of the imbalance of information supply and demand and cognitive overload

It is recommended that medical staff implement the following discharge education checklist according to the patient's primary cancer type, bone destruction site, education level, and family support system, combined with the discharge education framework (see Table 5):

**Table 5:** Discharge Education Framework.

Dimensions	Specific actionable measures (Examples)	Desired goals
Timing Personalization	Use “progressive education”: start basic knowledge on the 3rd day of admission, instruct specific skills on the 2nd day before discharge, and check only the key checklist on the day of discharge.	Reducing “cognitive load” on discharge day (Theme Large amount of information and poor satisfaction)
Form Personalization	Provide “short video + graphic manual” for those with low education; And “mobile APP+ in-depth science popularization” for those with high education.	Overcoming “Barriers to Understanding Medical Terms” (Theme Barriers to understanding medical terms)
Content Personalization	Create a “SREs Home Emergency card” that includes a 24-hour supervisor hotline and the nearest emergency route.	Alleviating “Coping with the demands of daily life” (Theme Coping with the demands of daily life)

Knowledge and skills checklist [31–34]: warning of SREs: identification of signs of pathological fracture (such as sudden severe pain and limb deformation). Medication safety: the three-step principle of analgesic drugs and monitoring the side effects of bone protective agents (such as bisphosphonates/denosumab). Personalization module: For bone damage parts, such as the spinal metastasis worn to roll over and support key guide axes, for patients with bone metastases of the lower extremities, nonweight-bearing transfer techniques were used. With respect to the primary disease, the module should be supplemented with respiratory function exercise information for lung cancer patients and should focus on the prevention of hormone-related bone loss for prostate cancer patients.

Psychosocial support list [3,35]: Family synchronization: Caregivers at home are responsible for aiding the patient, turning the patient over when needed, supporting the patient in relieving themselves, and ensuring a safe environment for the patient (e.g., installation armrest, nonslip handle). Dignity and social participation: Provide a “patient support group” or remote psychological consultation entrance for patients with a social withdrawal tendency to reduce their “burden”.

## Conclusion

Providing health education is crucial for medical personnel to ensure that individual hospital health education plans according to patient requirements, provides patients with bone health information and self-management behaviour guidance, and focuses on patients’ psychological distress while maintaining continued support for bone health after discharge.

## Limitations of the study

Although this study provides insights into the discharge health education needs of patients with solid tumour bone metastases and SREs, the following limitations still exist: Sampling bias and sample representativity. First, a single-centre design was used in the study, and the sample size was relatively limited, which may limit the extrapolation of the findings. Second, the demographic diversity of the sample is limited, as the number of participants in urban areas and those with a high level of education is significantly greater for men than for women, which may explain why the results cannot fully reflect the real experience of patients residing in rural areas or patients with lower levels of education. Additionally, this study is only from the perspective of patients and lacks the perspective of caregivers (such as family members and nursing workers). In the context of Chinese familism culture, caregivers play a crucial and often decisive role in the decision-making and daily care of patients after discharge. A lack of their feedback may lead to a lack of integrity in the discharge health education plan at the family implementation level. Finally, the interviews focused on specific time points before discharge. However, patients’ needs for bone health management and their actual experience changed dynamically with the progression of disease and the extension of discharge time. It was difficult to capture the new adaptive barriers or information needs of patients that may occur after a long period of discharge, such as 3–6 months, with the current design.

## Supporting information

S1 File Encoding Tree. Encoding tree of the qualitative data analysis.
(PDF)

S2 File COREQ checklist. Completed COREQ (COnsolidated criteria for REporting Qualitative research) checklist.
(DOCX)
