# Supplementary material for: Discharge health education needs and experiences of patients with skeletal-related events from bone metastases: A qualitative study
Source: PLoS One. 2026 Jun 12;21(6):e0341790. doi: 10.1371/journal.pone.0341790 (PMC13262820; doi:10.1371/journal.pone.0341790)
Supplement: S1 File — Encoding tree of the qualitative data analysis. (PDF) [file pone.0341790.s001.pdf]

Encoding tree

- Information experience
  - Obstacles in understanding medical terms
    - medical terms lead to confusion and panic
      - Lack of understanding of the medical term "skeletal-related event"（p2）
      - Feeling confused due to the excessive use of medical terms（p2）
      - Feeling powerless due to the difficulty in understanding medical terms（p14）
      - Feeling panicked because of not understanding medical terms（p13）
      - Difficulty understanding information due to medical terminology（p5）
    - Misunderstanding the doctor-patient relationship due to medical terms
      - Due to not understanding the purpose of the review items, there was a misunderstanding and lack of trust towards the doctor（p14）
    - Can't remember the names of the drugs and their side effects
      - Unable to remember the names of the drugs and their side effects, resulting in confusion due to medical terms（p2）
    - Desire for clear and understandable communication
      - Desire for an easy-to-understand communication method（p13）
  - Passive acceptance will not enable one to obtain targeted information
    - Don't ask questions or interrupt
      - Because of the doctors' professional authority, one is afraid to ask questions and can only passively accept（p2）
      - Worried about being judged negatively for asking questions, finding oneself in a dilemma.（p13）
      - Due to the doctor's professional authority, one is reluctant to express confusion and instead passively endures it.（p5）
    - Passively listening to the lecture, I felt that the content was useless and didn't dare to interrupt
      - Passively listening to the lecture, I felt that the content was useless and didn't dare to interrupt（p2）
    - The specific questions remain unanswered.
      - The specific questions remain unanswered and the information lacks relevance.（p8）
      - The questions regarding the side effects of the medication have not been answered（p13）
  - The large amount of information leads to low satisfaction.
    - Information overload, unable to remember
      - The excessive amount of information makes it difficult to remember and causes fatigue（p2）
      - There is too much information and it's impossible to remember it all.（p2）
      - Desire to learn, but there is too much information and it's difficult to remember（p14）
      - Long-term guidance that is hoped to continue, such as group chats on WeChat, etc（p10.14）
    - The timing for information dissemination is inappropriate, and both the body and mind are exhausted
      - The timing for information dissemination is inappropriate, and both the body and mind are exhausted（p14）
    - Due to the rushed discharge, the experience was not very satisfactory and I was unsure about it
      - Due to the hasty discharge, the information dissemination was rushed and the experience was poor, leaving one feeling uncertain（p13）
      - When one is unwell, they are forced to absorb a large amount of information, which leads to forgetfulness and a sense of unease（p10）
- Discharge health education needs
  - Knowledge requirements regarding bone health
    - Acquire basic knowledge of the skeleton
      - Desire to acquire basic knowledge about protecting the bones（p4）
    - Learn about the knowledge related to bone metastasis
      - Lack of knowledge about bone metastasis, hoping to learn about it（p6）
  - Self-management needs for bone health
    - Get long-term, ongoing guidance
      - Hope to obtain long-term and continuous guidance on bone health management（p4）
      - Desire for constant home guidance（P6）
      - It is hoped that it will be easy to contact medical personnel when needed（P2）
    - Practical management of the home
      - Hope to obtain practical management methods at home（p2）
      - The need for practical knowledge of home management was emphasized（p2）
    - Medication instructions and reminders
      - Medication confusion, need specific medication guidance and reminders（P4）
      - Misconceptions about the use of painkillers（p3）
    - Rehabilitation activity guidance
      - Seeking professional guidance on returning to daily activities and sports(p12)
    - Regular review of guidance
      - Specific instructions for review are needed（p4）
    - Prevention of falls
      - Activity-induced fear, requiring preventive guidance for activities to avoid falls（P9）
  - Addressing daily life needs
    - Adjustment of daily habits
      - Need suggestions on lifestyle adjustments such as drinking habits（p1）
      - Need guidance on nutritional supplementation（p11）
      - Need guidance on daily activities such as turning over and using the toilet（p7）
  - Dealing with the need for psychological stress relief
    - Feeling guilty due to relying on others
      - Feeling painful and guilty because everything depends on others（p2）
    - Hopelessness due to illness
      - Feelings of hopelessness due to loss of physical function and dependence on others（p3）
    - The need to preserve the dignity of the individual
      - Suffering from being a burden to the family and longing for personal dignity（p9）
      - Desire to be respected and maintain the dignity of an elder（p1）
  - Access to family and social support needs
    - Desire for emotional connection and enlightenment
      - Longing for family guidance and emotional connection（p15）
    - Seek mutual assistance and a sense of belonging among patients
      - Seeking mutual assistance and sense of belonging of patients（p6）
      - They were eager to join the patient group and get emotional support（p15）
    - Worry about social discrimination and isolation
      - Worry about social discrimination, feel lonely（p6）
    - Medical concealment, (family protection)
      - They chose to hide it for fear of increasing the burden on their family（p1）
      - To suffer alone so as not to upset your family（p1）
      - Fear of becoming a nuisance and concealing illness and pain（p1）
